# Bispecific Antibody Affords Complete Post-Exposure Protection of Mice from Both Ebola (Zaire) and Sudan Viruses

**DOI:** 10.1038/srep19193

**Published:** 2016-01-13

**Authors:** Julia C. Frei, Elisabeth K. Nyakatura, Samantha E. Zak, Russell R. Bakken, Kartik Chandran, John M. Dye, Jonathan R. Lai

**Affiliations:** 1 Department of Biochemistry, Albert Einstein College of Medicine, 1300 Morris Park Ave., Bronx, NY 10461; 2 Virology Division, United States Army Medical Research Institute of Infectious Disease, 1425 Porter Street, Fort Detrick, MD 21702; 3 Department of Microbiology and Immunology, Albert Einstein College of Medicine, 1300 Morris Park Ave., Bronx, NY 10461.

## Abstract

Filoviruses (Ebola and Marburg) cause severe hemorrhagic fever. There are five species of ebolavirus; among these, the Ebola (Zaire) and Sudan viruses (EBOV and SUDV, respectively) are highly pathogenic and have both caused recurring, large outbreaks. However, the EBOV and SUDV glycoprotein (GP) sequences are 45% divergent and thus antigenically distinct. Few antibodies with cross-neutralizing properties have been described to date. We used antibody engineering to develop novel bispecific antibodies (Bis-mAbs) that are cross-reactive toward base epitopes on GP from EBOV and SUDV. These Bis-mAbs exhibit potent neutralization against EBOV and SUDV GP pseudotyped viruses as well as authentic pathogens, and confer a high degree (in one case 100%) post-exposure protection of mice from both viruses. Our studies show that a single agent that targets the GP base epitopes is sufficient for protection in mice; such agents could be included in panfilovirus therapeutic antibody cocktails.

The family *Filoviridae* (“filoviruses”) includes five species of ebolavirus and Marburg virus (MARV). Infection by these negative-stranded RNA viruses causes severe hemorrhagic fever with human case fatality rates as high as 90%^1^,^2^. Filovirus outbreaks are sporadic in nature and, prior to 2014, were limited to fewer than 500 cases^3^. However, the 2014 epidemic in West Africa, still on-going in some regions, is unprecedented in terms of total size (over 28,000 suspected cases as of this writing), geographic distribution, and longevity. The five species of ebolavirus are named for the regions in which they were first identified: Zaire (EBOV), Sudan (SUDV), Bundibugyo (BDBV), Tai Forest (TAFV), and Reston (RESTV)^2^. The West Africa epidemic is caused by a novel isolate of EBOV (Makona)^4^,^5^,^6^. Based on the history of human outbreaks, there appears to be a broad distribution of pathogenicity and geographic location for EBOV, SUDV, and BDBV, the three species that have caused recurring, large outbreaks. EBOV and BDBV outbreaks have generally been associated with Central Africa, mostly in the Democratic Republic of Congo, and SUDV outbreaks with Uganda and South Sudan. The only previous case of any filovirus in West Africa, prior to 2014, was a single case of TAFV in 1994. These facts highlight the unpredictable nature of filovirus outbreaks and underscore the potential benefits of cross-species vaccines and therapeutics. Recently, a vesicular stomatitis virus-vectored EBOV vaccine was shown to be 100% effective in a limited clinical trial in Guinea^7^. Although highly encouraging, there is still a strong need for broad therapeutics for post-exposure treatment in cases of unvaccinated individuals or where the vaccine cannot be provided.

There are a number of therapeutic filovirus platforms under development including several with demonstrated efficacy in non-human primate (NHP) models^8^,^9^. Monoclonal antibody (mAb) cocktails and convalescent IgG therapies are particularly attractive options, owing to the generally favorable pharmacokinetic properties of antibody therapeutics^10^,^11^,^12^,^13^,^14^,^15^,^16^,^17^. Both mAb cocktails and convalescent IgG have been shown to provide post-exposure protection to NHPs against EBOV and, in the case of convalescent IgG, MARV. In addition, one antibody cocktail known as ZMapp (Mapp Biopharmaceutical) was provided on a compassionate basis in several human cases in 2014, is currently in clinical trials, and has been shown to reverse the course of Ebola virus disease (EVD) five days post-infection in non-human primates^8^,^13^. Although not as advanced, protective mAbs have now also been described for MARV and SUDV^10^,^11^,^14^,^18^,^19^. Several recently reported MARV human mAbs exhibit cross-reactivity for the EBOV glycoprotein (GP) core^11^,^20^.

The glycoprotein GP is the sole protein on the virus surface, is the primary target of neutralizing antibodies, and is required for entry into host cells^12^,^15^,^20^,^21^,^22^,^23^,^24^. The prefusion GP spike consists of three copies of the two subunits, GP1 (the surface subunit) and GP2 (the transmembrane or fusion subunit). Three-dimensional structures, determined by X-ray crystallography, have been reported for EBOV, SUDV, and MARV prefusion GP, and for the EBOV and MARV GP2 fusion subunit in the post-fusion conformation^15^,^20^,^21^,^22^,^25^,^26^,^27^. EBOV and SUDV GP prefusion structures are similar and consist of a trimeric chalice-and-bowl morphology, with the GP2 subunits forming the chalice on top of which the GP1 subunit trimer forms the bowl. Both atomic resolution (X-ray crystallography) and lower resolution (cryoelectron microscopy) structural studies of GP-antibody complexes have suggested that the GP “base” epitope, at or near the interface of GP1 and GP2 in the prefusion form, is particularly susceptible to neutralization by antibodies (Fig. 1a). EBOV neutralizing mAbs KZ52 (human), 4G7 and 2G4 (both ZMapp components) as well as SUDV mAb 16F6 (murine) and humanized variants thereof target this region^12^,^14^,^15^,^20^,^21^,^22^,^28^. Additional components of ZMapp and related cocktails include antibodies that bind in the glycan cap or mucin-like domain of GP1, and whose precise function is not clear^12^,^29^. Nonetheless, the structural and protective data on EBOV mAbs suggest that engagement of multiple epitopes, including at least one component directed at the GP base, is an important feature of therapeutic mAb cocktails.

Both EBOV and SUDV have caused outbreaks exceeding 300 cases, and are associated with average human case fatality rates of 70% and 52%, respectively, from 1976–2012. We sought to develop novel antibodies that could cross-neutralize and provide cross-protection in animals for these two species. Cross-reactive human polyclonal antibodies from vaccination of transchromosomal cattle were recently demonstrated to provide post-exposure protection against both EBOV and SUDV in mice;^30^ and murine mAbs obtained from co-immunization with EBOV and SUDV GPs also provided partial protection of mice against both viruses with multiple doses^19^. These data suggest that protection against multiple species is possible in mice. However, only a handful of cross-reactive mAbs exist and none have been demonstrated to provide post-exposure protection against multiple filoviruses in animals^18^,^20^,^31^,^32^,^33^. Despite similar overall structural features of the prefusion assembly, and a similar susceptibility site at the GP base epitope, the amino acid sequences between EBOV and SUDV GPs are significantly divergent (45%), which is the likely reason that cross-neutralizing antibodies against these two species are rare. For filoviruses and other viruses, cocktails of mAbs that engage multiple susceptible epitopes are likely to be most efficacious; therefore, there is a significant motivation for simplifying potential cross-neutralizing cocktails by development of single component, cross-neutralizing mAbs. As a step toward this goal, we report the engineering and *in vitro* characterization of bispecific antibodies that can neutralize both EBOV and SUDV, and afford complete post-exposure protection in mice.

## Results

### Design and Binding Profiles of Bispecific Antibodies (Bis-mAbs)

**Figure 1:**
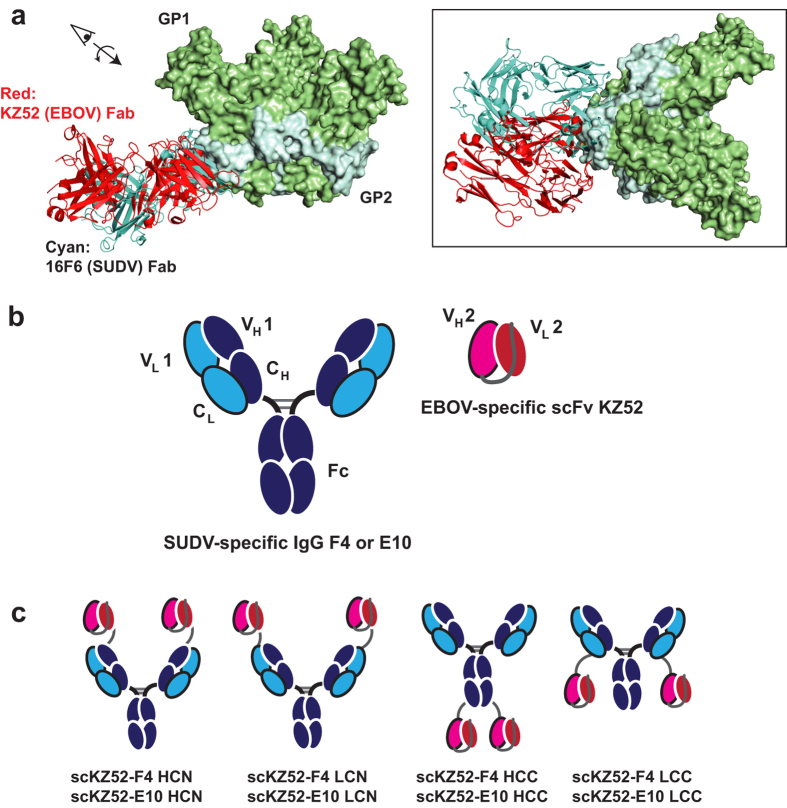
Design of bispecific antibodies. KZ52 is a human EBOV mAb, and F4 and E10 are synthetic human scaffold SUDV mAbs. (**a**) The binding sites of KZ52 on EBOV GP (PDB ID: 3CSY) and 16F6, the murine parent of E10 and F4, on SUDV GP (PDB ID: 3S88) are similar and involve a structural epitope at the base of the prefusion trimer^15^,^22^. (**b**) Bis-mAbs described here consist of fusions between the KZ52 scFv (V_H_-V_L_ topology) and the IgG of either F4 or E10. (**c**) Several fusion topologies were examined: heavy chain N-terminus (HCN), light chain N-terminus (LCN), heavy chain C-terminus (HCC), and light chain C-terminus (LCC).

A number of design modalities exist for creation of altered IgG scaffolds with the capacity to engage multiple antigens^34^. As a preliminary evaluation, we chose to create simple single chain variable fragment (scFv)-IgG fusions containing variable domains for EBOV-specific human mAb KZ52 and the SUDV-specific humanized mAbs E10 and F4 (Fig. 1b)^14^,^22^. KZ52 was isolated from a human survivor and has been shown to provide post-exposure protection as a single mAb therapy in guinea pigs and mice, but in a single study did not protect NHPs^35^,^36^,^37^. Nonetheless, KZ52 was used as an initial test case for EBOV-targeting Bis-mAbs because it is of human origin, and because the high resolution three-dimensional structure is available, which allowed us to accurately predict the beginning and end points of the structured variable domains. SUDV mAbs E10 and F4 were derived from synthetic libraries based on the Herceptin (4D5) scaffold with altered complementarity-determining region (CDR) segments from the murine SUDV mAb 16F6, and have been demonstrated to provide post-exposure protection of immunocompromised mice^14^. A KZ52 scFv of V_H_-V_L_ topology with a (GGGS)_3_ linker was fused genetically to the IgGs of E10 and F4 via a ~GGSASGSAGSAGSGGS~ linker sequence to create the preliminary Bis-mAb panel shown in Fig. 1c. Several locations of the scFv were evaluated, at the N-terminus of the light chain (“LCN”) or heavy chain (“HCN”), and at the C-terminus of the light chain (“LCC”) and heavy chain (“HCC”). All constructs expressed readily in HEK293F or HEK293T suspension cells and could be purified by Protein A affinity chromatography. The “LCC” constructs were susceptible to degradation, presumably by proteolysis, but this could be mitigated by addition of protease inhibitors. Size-exclusion chromatography coupled with multiangle light scattering (SEC-MALS) analysis of constructs scKZ52-F4 HCC and scKZ52-F4 LCN indicated a monodisperse population of monomers, with some higher aggregate present (Supplementary Figure 1). Purified Bis-mAbs of the LCN, HCC, and LCC domain organization were found to be stable at 4 °C for one year with moderate degradation as analyzed by SDS-PAGE (Supplementary Figure 2); we tested the full binding and neutralization profile of a 10-month old sample of scKZ52-E10 HCC and found these features to be unaffected by extended storage under these conditions.

**Figure 2:**
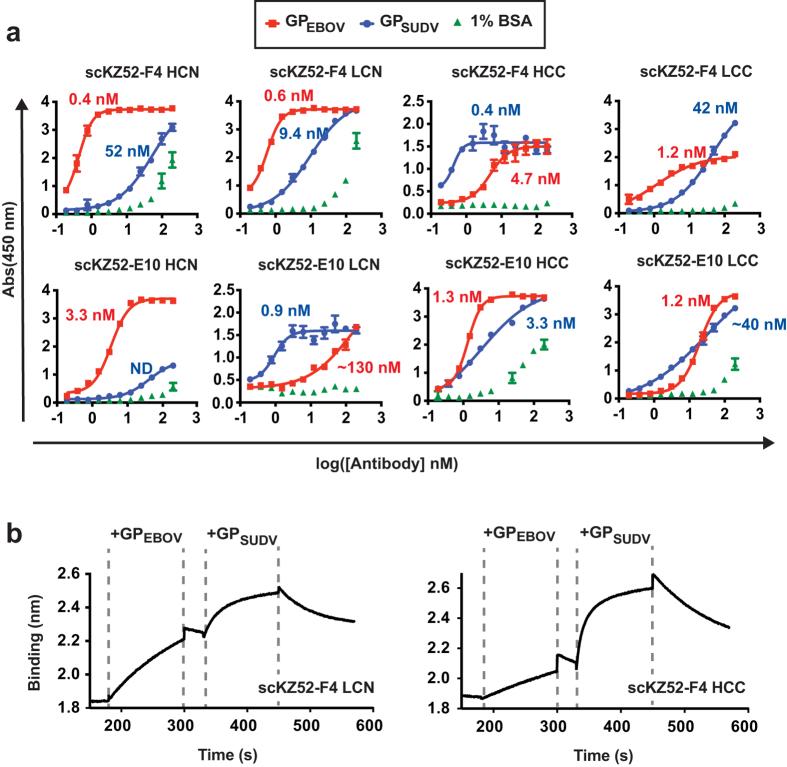
Binding profiles. (**a**) ELISA reactivity of the Bis-mAbs for GP lacking the transmembrane domain from EBOV and SUDV were examined. The numerical half-maximal binding titer (EC_50_) is listed next to each curve. Wells coated with 1% BSA were included as a control for non-specific binding. (**b**) Two-phase binding experiments for scKZ52-F4 LCN and scKZ52-F4 HCC by biolayer interferometry. The Bis-mAb was loaded onto the probe, and then dipped sequentially into analyte solutions containing GP_EBOV_ then GP_SUDV_.

Bis-mAbs were evaluated for reactivity toward the glycoprotein lacking the transmembrane domain from EBOV (Kikwik, GP_EBOV_) and SUDV (Boniface, GP_SUDV_) by ELISA (Fig. 2a). In general, most constructs exhibited strong cross-reactivity with half-maximal binding titers (EC_50_s) ranging from 0.4–130 nM. Binding of Bis-mAbs to GP_EBOV_ was strong, with EC_50_s in the low nanomolar or subnanomolar range for all constructs except scKZ52-E10 LCN, but somewhat diminished relative to monospecific KZ52 (0.01 nM, Supplementary Figure 3). The EC_50_ values for GP_SUDV_ of the LCN and HCC domain organization were similar to previously reported values for monospecific IgGs F4 and E10 (3.9 and 2.1 nM, respectively)^14^. However, binding to GP_SUDV_ was significantly lower for the HCN and LCC constructs relative to their parental monospecific IgGs. Cross-reactivity of Bis-mAbs to negative control wells coated with 1% BSA was observed in several cases, potentially due to unfolding tendencies of the scFv fragments, but the onset of this non-specific binding activity was not until much higher antibody concentrations (>100 nM). Notably, scKZ52-F4 HCC, scKZ52-F4 LCC, scKZ52-E10 HCN and scKZ52-E10 LCN exhibited no cross-reactivity toward BSA. The EC_50_ values for GP_EBOV_ and GP_SUDV_ were within ~20-fold of one another for most Bis-mAbs, except for the “HCN” constructs, which had markedly lower activity for the GP_SUDV_ and scKZ52-E10 LCN which had lower activity for GP_EBOV_. The disparity for the “HCN” constructs is likely because fusion of the KZ52 scFv to the N-terminus of the heavy chain occluded critical epitope-contacting residues in the E10 and F4 V_H_ domain. The structures for E10 and F4 are not available, but they are closely related to and compete with murine mAb 16F6 for binding to GP_SUDV_, which requires extensive interactions between CDR-H1 and CDR-H3 and the GP epitope^15^.

The binding affinities of scKZ52-F4 LCN and scKZ52-F4 HCC were characterized by biolayer interferometry (BLI, Fig. 2b and Supplementary Table 1). Modeling of initial association and dissociation phases in single-phase binding experiments with scKZ52-F4 HCC to GP_EBOV_ or GP_SUDV_ provided dissociation constants (K_D_s) of 240 nM for GP_EBOV_ and 170 nM for GP_SUDV_. The observed affinity for GP_SUDV_ of the Bis-mAb is similar to affinity estimates of the canonical F4 IgG1s by competition ELISA (100–200 nM), indicating that the scFv fusion did not alter the affinity of the combining site. However, the affinity of the Bis-mAbs for GP_EBOV_ was significantly lower than has been reported for the KZ52 IgG1 by surface plasmon resonance (K_D_ = 6.3 nM), likely because conversion from the IgG to the scFv format compromises structural integrity of the V_H_-V_L_ interface^38^. The capacity of Bis-mAbs scKZ52-F4 LCN and scKZ52- F4 HCC to engage both antigenic targets simultaneously was also examined in two-phase binding experiments. Immobilized Bis-mAb on the biosensor probe was first exposed to analyte solutions containing GP_EBOV_ followed by a short equilibrium phase and transfer to a solution containing GP_SUDV_ (Fig. 2b). The results demonstrate that both GP antigens can be engaged simultaneously.

### Virus Neutralization

**Figure 3:**
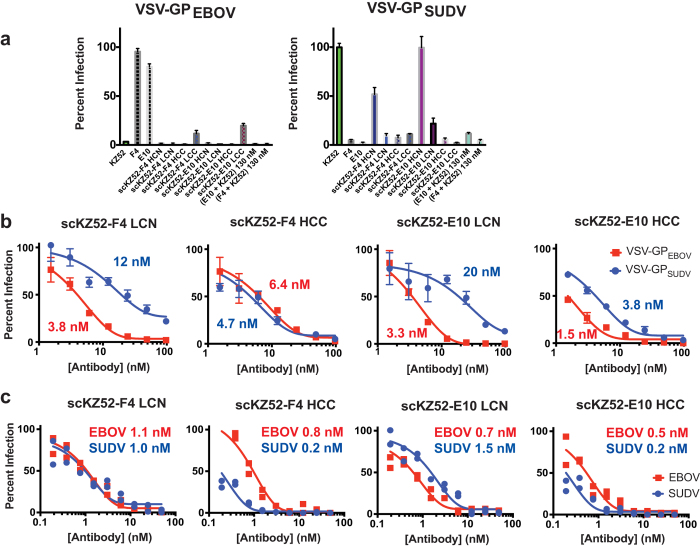
Virus neutralization. (**a**) Single-point neutralization assay of all Bis-mAbs at 100 nM against VSV-GP_EBOV_ or VSV-GP_SUDV_. KZ52, F4, and E10 were included as controls. In addition, equimolar mixtures of E10 + KZ52 and F4 + KZ52 at 130 nM (equivalent mass amounts to Bis-mAbs) were included for comparison. (**b**) Dose-dependent neutralization of VSV-GP_EBOV_ and VSV-GP_SUDV_ by scKZ52-F4 LCN, scKZ52-F4 HCC, scKZ52-E10 LCN, and scKZ52-E10 HCC. Calculated IC_50_ values are listed next to each curve. (**c**) Neutralization of authentic viruses by PRNT assay, with IC_50_ values listed.

The Bis-mAbs were tested for their capacity to inhibit viral entry in a single-round infectivity experiment using vesicular stomatitis virus pseudotyped with GP from EBOV or SUDV (VSV-GP_EBOV_ and VSV-GP_SUDV_) (Fig. 3a,b)^14^,^39^. In this assay, VSV-GP_EBOV_ or VSV-GP_SUDV_ particles bearing enhanced green fluorescent protein (eGFP) in the viral genome are used to infect a monolayer of Vero cells, and infection events are quantified by fluorescence microscopy. A preliminary high-point (100 nM) assay of all Bis-mAbs demonstrated potent neutralization, >90% in most cases, of VSV-GP_EBOV_ (Fig. 3a). All Bis-mAbs except the HCN constructs also exhibited strong neutralization against VSV-GP_SUDV_; the lack of HCN activity against VSV-GP_SUDV_ correlates with the reduced binding observed by ELISA. In contrast, the monospecific controls KZ52 (EBOV), and F4 and E10 (SUDV) had neutralizing activity against only the relevant pseudotyped virus. Furthermore, equimolar mixtures of E10 + KZ52 or F4 + KZ52 (130 nM total antibody concentration, which corresponds to an equivalent mass amount of Bis-mAb) provided similar neutralization of both pseudotyped viruses.

Based on favorable expression and stability profiles, Bis-mAbs scKZ52-F4 LCN, scKZ52-F4 HCC, scKZ52-E10 LCN, and scKZ52-E10 HCC were chosen for additional analysis. These four Bis-mAbs showed dose-dependent neutralization of both VSV-GP pseudotyped viruses, although the IC_50_ values were in general higher for VSV-GP_SUDV_ than VSV-GP_EBOV_ for the “LCN” constructs (Fig. 3b). For both “HCC” constructs the relative IC_50_ values for the two pseudotyped viruses were approximately the same. In all cases, the highest levels (≥95%) of neutralization were achieved at 100 nM Bis-mAb concentration against both pseudotyped viruses. The monospecifc controls were found to have IC_50_ values of 2.7 nM (KZ52 vs. VSV-GP_EBOV_), 3.5 nM (F4 vs. GP_SUDV_), and 7.0 nM (E10 vs. VSV-GP_SUDV_) (Supplemental Fig. 4), indicating that neutralization potential was generally comparable for VSV-GP_EBOV_ upon conversion of the KZ52 variable domains into a scFv. Modification of the F4 and E10 IgGs resulted in moderate reductions in activity when the KZ52 scFv was conjugated in the LCN format, but had no effect in the HCC format. The four Bis-mAbs were also tested for their capacity to neutralize authentic EBOV (Kikwit-1995) and SUDV (Boniface-2000) under BioSafety Level 4 (BSL4) conditions using a plaque reduction neutralization test (PRNT) (Fig. 3c). For all Bis-mAbs, potent neutralization of both EBOV and SUDV were observed in both assays, with low- or sub-nanomolar IC_50_ values in the PRNT assay for both viruses.

### *In Vivo* Efficacy

**Figure 4:**
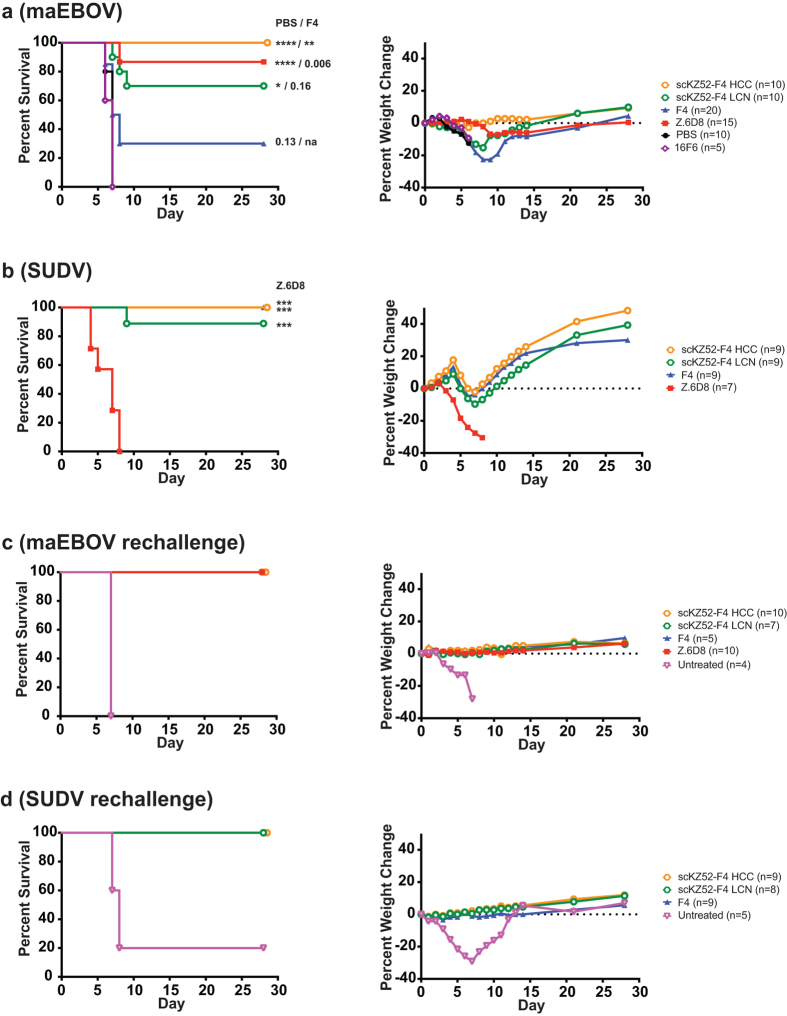
Animal challenge experiments. Percent survival and median percent weight change for mouse-adapted EBOV (**a**) and SUDV (**b**) challenges with antibody treatment. Mice were treated with a single post-exposure dose (200 μg at 24 hours) for EBOV or two post-exposure doses (500 μg at days +1 and +4) for SUDV. Statistical p-values are listed against PBS and F4 controls for EBOV and against Z.6D8 control for SUDV (****p < 0.0001; **p < 0.01; *p < 0.05; p-values greater that 0.05 are listed numerically; na, not applicable). (**c**,**d**) Rechallenge of surviving mice from initial challenge cohorts, with no further antibody treatment.

To explore the protective potential of a single cross-binding monoclonal antibody agent, scKZ52-F4 LCN and scKZ52-F4 HCC were evaluated for their ability to confer protection of mice in two separate mouse models with post-exposure dosing (Fig. 4). These two Bis-mAbs were chosen because the LCN and HCC domain organizations were the best performing in both binding and neutralization studies. SUDV mAbs F4 and E10 are highly similar in terms of properties, differing by only a few amino acid substitutions, and therefore the F4 backbone should be representative^14^. For EBOV, a well-established mouse challenge model was employed with mouse-adapted (ma) EBOV (Mayinga) and WT C57BL/6 mice^40^. For SUDV, we have previously reported a model for pathogenicity that utilizes type I interferon α/β receptor knockout mice infected at 4 weeks old with human lethal SUDV (Boniface)^14^,^41^. For maEBOV, a single Bis-mAb dose (200 μg) was provided 24 hours post-challenge and for SUDV, two doses (500 μg each) were provided at one and four days post-exposure. We note that the SUDV model, which involves immunocompromised mice infected at 4 weeks of age, provides a more stringent requirement for protection against initial viral challenge, and thus higher antibody doses are required than that used for the maEBOV model. Mono-specific mAbs Z.6D8 (EBOV) and F4 (SUDV) were included as controls as they have previously shown excellent protection against their respective viruses^14^.

Both Bis-mAb treatments resulted in high (>70%) protection in both models, with scKZ52-F4 HCC conferring 100% protection against both viruses. As expected, Z.6D8 as a treatment was not protective against SUDV but was highly protective (89%) against EBOV. SUDV monospecific mAb F4 provided 100% protection against SUDV, as we have previously reported^14^ and afforded partial (30%) protection against maEBOV. However, we note that the level of protection for F4 against maEBOV is not statistically distinguishable from a PBS control group, in which all mice succumbed to disease by day 7 (p = 0.13). Murine 16F6 afforded no protection against maEBOV in a group size of n = 5 challenge experiment. The 100% protection from maEBOV observed for scKZ52-F4 HCC was statistically distinguishable from both PBS and F4 controls in this experiment, whereas 70% protection of scKZ52-F4 LCN was distinguishable only from the PBS. These results indicate that both scKZ52-F4 HCC and scKZ52-F4 LCN provide statistically significant protection relative to a PBS control group. For SUDV, both Bis-mAbs and F4 were significantly protective relative to the Z.6D8 negative control treatment group.

Aggregate weight loss was observed in the Bis-mAb- and F4-treated group during the course of the SUDV challenge, a phenomenon we have previously reported for post-exposure dosing of protective SUDV mAbs^14^,^41^. However, the surviving Bis-mAb or mAb-treated population continued to gain weight (on average) after day 8. For maEBOV, mean weight loss was observed during the initial infection period for scKZ52-F4 LCN- and F4-treated mice, as some mice became sick, but this trend was not observed with either Z.6D8 or scKZ52-F4 HCC mice. All mice from the Z.6D8- and scKZ52-F4 HCC-treated groups survived the infection.

To examine the capacity for memory immunity in Bis-mAb and mAb-treated mice, the surviving cohort of both EBOV and SUDV challenges were subjected to rechallenge (with the same isolate of virus) without mAb treatment 35 days after the initial challenge (Fig. 4c,d). For both EBOV and SUDV, Bis-mAb or mAb treated mice were completely protected against viral rechallenge with no observable aggregate weightloss, indicating memory immunity had been established. In both cases, an untreated negative control group was also included to confirm lethality of the virus.

## Discussion

We describe here the design and characterization of Bis-mAbs with potent neutralization activity against both EBOV and SUDV. Viral therapeutic bispecific antibody modalities have previously been described to target multiple serotypes of Dengue virus, or simultaneously engage host and viral components for HIV-1^42^,^43^; the work described here represents the first use of this approach to develop cross-neutralizing filovirus antibodies. Since EBOV and SUDV have collectively accounted for 95% of Ebola-related deaths from 1976–2012, these two viruses were chosen as an initial test case for development of cross-neutralizing Bis-mAbs. Both components of the Bis-mAb design contain human or humanized scaffolds, and therefore this particular combination of antibodies may be desirable for therapeutic purposes. Although there is potential for therapeutic synergy of designed Bis-mAbs, alone or as components of cocktails, relative to traditional antibodies, this has not been tested explicitly here. The studies herein provide proof-of-concept that an antibody with two binding modalities (KZ52 and F4/E10 Fvs) is sufficient to confer high levels of post-exposure protection in mice against EBOV and SUDV. The effectiveness of other combinations of antibodies targeting other regions of GP, and whether they provide enhanced protective ability remains to be determined. While protection of mice provides a good initial criterion, evaluation in larger animal models will be required to fully assess the potential of any broad filovirus immunotherapy.

It is noteworthy that complete (scKZ52-F4 HCC) or near-complete (scKZ52-F4 LCN) cross-protection could be afforded by genetic linkage of two mAbs recognizing the GP base epitope. Two of the EBOV cocktail ZMapp components (c2G4 and c4G7) bind in this region, although with different angles relative to the plane of the viral membrane^12^. In addition, the atomic features for recognition by KZ52 (EBOV) and 16F6 (SUDV), for which X-ray structures are available, indicate that there are some structural differences between the epitopes^15^,^22^. Nonetheless, it appears that binding the GP base epitope is important for neutralization and confers protection at least in rodent models for these two filoviruses. Our studies establish that a single agent with specificities toward base epitopes of two GPs can afford this cross-protective capability. Whether or not other filoviruses will be susceptible at this epitope is still unknown; structural studies on the MARV GP suggest that this region may be occluded by the mucin-like domain, which is predicted to sit lower on the prefusion spike than on EBOV or SUDV^20^. Nonetheless, our work suggests that cross-neutralization efforts should focus on the GP base epitope, and that cross-species protection can be achieved by a single agent for at least these two filoviruses.

Although the precise requirements for GP epitopes and protection in larger animals has not been established across filovirus species, for antiviral immunotherapies in general, it seems likely that targeting of multiple epitopes within a mAb cocktail would be advantageous. Engagement of multiple epitopes allows for multiple potential mechanisms of protection, for example, non-neutralizing mAbs can also provide protection through Fc-mediated functions. In addition, binding to multiple epitopes reduces the risk of escape by a single mutation. For EBOV, protection of NHPs by a single mAb has not been demonstrated, and for SUDV, protection of NHPs by mAbs in general has not been shown. Since cross-neutralizing mAbs for EBOV and SUDV are rare, a potential strategy to reduce complexities of cross-protective cocktails is the combination of two or more binding specificities by antibody engineering. Here we demonstrate that this approach provides complete protection in one case for mice. The Bis-mAb format described here has therapeutic potential, but other formats that do not employ long polypeptide linkers, which are susceptible to proteolysis or that may lead to immunogenicity, would be desirable for a cross-neutralizing mAb targeting this region.

## Methods

### Antibody Expression and Purification

The synthetic genes for all Bis-mAbs were obtained from a commercial supplier (Genewiz, Plainview, NJ) and subcloned into pMAZ-IgL and pMAZ-IgH vectors^44^. Vectors for the heavy and light chain were transfected into HEK293F cells (Invitrogen, Grand Island, NY) using 2 μg/mL linear polyethylenimine (PEI) molecular weight 25,000 Daltons according to the manufacturer’s instructions (Polysciences, Warrington, PA). Cell cultures were incubated at 37 °C and 8% CO_2_ for 5–6 days post-transfection. The cell cultures were centrifuged and the supernatants were applied to a protein-A affinity column (~1 mL packed beads per 600 mL culture) (Pierce, ThermoScientific, Rockford, IL). Bis-mAbs were purified using the Gentle Antibody Elution System (Pierce, ThermoScientific, Rockford, IL) as per manufacturer’s protocol. Antibodies were desalted into 150 mM HEPES and 200 mM NaCl pH 7.4 for use in experiments.

Antibodies used in mouse challenge studies were produced under endotoxin-free conditions by KempBio, Inc. (Frederick, MD). HEK293T cells in serum-free suspension culture were cultivated in shake-flasks incubated at 37 °C with an atmosphere composed of 95% Air + 5% CO_2_. The culture medium was FreeStyle 293 (ThermoFisher Rockford, IL) supplemented with 2 mM L-glutamine and 50 mg/L G418 (both ThermoFisher, Rockford, IL). The plasmids used for each transfection were amplified in DH5-α cells (ThermoFisher, Rockford, IL) and purified using an endotoxin-free kit from Macherey Nagel. The HEK293T cells were transfected in log-phase growth when the cell density was between 1 and 1.5 × 10^6^ cells per mL. Equal amounts of each plasmid were combined to yield a total of 1 milligram of plasmid DNA per liter of culture. Each milligram of DNA was complexed with 4 milligrams of PEI-Max (MW 40000, Polysciences, 1 mg/mL in saline) and incubated for 5 minutes at room temperature prior to addition to the cell cultures. The cultures were maintained as described above for 120 hours and the culture supernatant containing the recombinant antibody was harvested and filter sterilized. Protein purification was performed using Protein A resin (MabSelect, GE Healthcare Life Sciences) and polished using Capto-Adhere resin (GE). The level of aggregation was assessed using size exclusion chromatography (S-200 Sephacryl 16/60, GE) and the endotoxin level was determined using the ToxinSensor Kit (GenScript, Piscataway, NJ). The final buffer for each antibody was PBS pH 7.4 (ThermoFisher, Rockford, IL) and the final protein level was assayed using A_280_ with an extinction coefficient of 1.4. The product was filter sterilized and stored at −20 or 2–8 °C.

### ELISA Assays

GP proteins expressed from HEK293 cells were purchased as previously described from the Protein Expression Laboratory, Frederick National Laboratory for Cancer Research^14^. The GP target proteins were directly immobilized onto 96-well Maxisorp plates (GP_SUDV_ and GP_EBOV_ = 0.5 μg/well) by incubating in PBS pH 8.0 for 14–16 hours at 4 °C. PBS, pH 7.4, containing 1% BSA was used to block the wells after target immobilization (incubation for 90–120 minutes at RT). Negative control plates were coated with 1% BSA only. Bis-mAbs were diluted into PBT (PBS pH 7.4, 1% BSA, and 0.05% Tween-20), applied to the wells, and incubated at RT for 1 hour. The plates were washed with PBST (PBS pH 7.4 and 0.05% Tween-20) and incubated for 60 minutes with Protein A/HRP antibody conjugate (1:1000 dilution in PBT). The wells were washed 4–6 times with PBST, developed using 3, 3′, 5, 5′-tetramethylbenzidine (TMB; Sigma-Aldrich, St. Louis, MO), and quenched with 0.5 M sulfuric acid. The absorbance at 450 nm was determined. The data was fit to standard four-parameter logistic equations using GraphPad Prism (GraphPad Software, La Jolla, CA). The half-maximal binding titers (EC_50_) were obtained from the inflection point in the curves.

### BioLayer Interferometry

The forteBio BLItz system was used to determine the binding properties of antibodies to GP_EBOV_ and GP_SUDV_. Anti-human Fc capture sensors were used for initial antibody loading, which was followed by GP_EBOV_ or GP_SUDV_ protein association and dissociation. Several different GP_EBOV_ or GP_SUDV_ concentrations, ranging from 0.125 μM to 3.6 μM, were used. For single-phase binding experiments, global data fitting to a 1:1 binding model was used to estimate the *k*_a_ (association rate constant), *k*_d_ (dissociation rate constant) and K_D_ (equilibrium dissociation constant) values. For double-phase binding experiment, antibody was immobilized first on sensor, and then allowed to equilibrate in a solution containing one of the GPs. The sensor was then transferred to a second solution containing the other GP.

### Pseudotyped Virus Neutralization Assays

Neutralization assays were performed using vesicular stomatitis virus pseudotyped to display the GP from either SUDV or EBOV in place of its native G glycoprotein (VSV-GP_SUDV_ or VSV-GP_EBOV_, respectively)^14^,^39^. The viral genome encodes an enhanced green fluorescent protein (eGFP) so that infection is scored by counting fluorescent cells after infection. The protocol for VSV-GP production has been described elsewhere^45^. Briefly, the virus-containing supernatants were harvested and concentrated by pelleting through a 10% sucrose cushion. Virus stocks were titered by infecting African Green Monkey kidney (Vero) cells with serial dilutions and counting eGFP-positive cells by fluorescence microscopy. VSV-GP was used to infect Vero cells at approximate multiplicities of infection of 0.1 to 1.0 in Dulbecco’s Modified Eagle Medium (DMEM) containing 2% fetal bovine serum (FBS; Thermo Scientific, Waltham, MA), such that 20–200 cells were infected per well. Vero cell monolayers consisting of ~7.5 × 10^4^ cells/well in a 48 well plate were incubated for 14–16 hours with pseudotyped virus that had been pre-incubated for 1 to 2 hours with dilutions of the Bis-mAb. Infection was scored by manually counting eGFP-positive cells under a fluorescence microscope, 14–16 hours after initial exposure. Virus was added to a well containing media alone (no antibody) to serve as a control for 100% infection.

Alternatively, a 96-well format was adopted to facilitate rapid generation of dose-dependent curves for neutralization. Vero cell monolayers were seeded at ~2.0 × 10^4^ cells/well in a 96 well plate and grown to confluency overnight at 37 °C. Pseudotyped viruses that had been pre-incubated for 1 hour with dilutions of Bis-mAb were incubated with the cells for 14–16 hours in DMEM supplemented with 2% FBS at 37 °C. Cells were then fixed using formaldehyde and stained with Hoechst 33342 Trihydrochloride, Trihydrate staining solution (ThermoFisher, Rockford, IL). Infection was then scored using the Thermo Scientific Cell Insight CX5 platform microscope and software that scores infection based on percentage of infected cells ([# of GFP positive cells/# of Hoechst positive cells]*100%). Virus added to wells with no antibody was used as a control for 100% infectivity, and wells with no virus and no antibody were used as 0% infection.

### Plaque reduction neutralization test with authentic pathogens

Dilutions of the antibody of interest were made in a sterile 96 well plate (Costar/Corning Incorporated, Corning, NY) in Eagle Minimum Essential Media (EMEM) (Sigma Aldrich, St. Louis, MO) supplemented with 5% FBS. In sterile 6 well plate (Costar/Corning Incorporated, Corning, NY), 125 μL of authentic EBOV or SUDV diluted to 1200 pfu/mL were added to each well and the plates were incubated at 37 °C for 1 hour. Virus was added to a well containing media alone (no antibody) as a control for 100% infection. Vero-E6 cells were exposed to 100 μL of the virus/antibody mixture and incubated at 37 °C for an additional hour. During this time, the plates were gently rocked every 15 minutes to ensure homogeneity and prevent drying. After 1 hour of incubation, 2 mLs of primary overlay (EMEM with 10% FBS and 1% Gentamicin (Sigma Aldrich, St. Louis, MO) with 1% SeaKem ME agarose (Lonza, Cohasset, MN)) was added to each well and the plates were incubated at 37 °C for 6 days. On Day 7 post-exposure to virus, neutral red solution (EMEM with 10% FBS and 1% Gentamicin with 5% neutral red (Gibco/Invitrogen, Grand Island, NY)) was added to all cell-containing wells and cells were incubated at 37 °C overnight. Infection was scored by counting the number of plaques per well, using the number of plaques on the control well (no antibody) as 100% infection.

### Mouse Challenge Experiments

Research was conducted under an IACUC approved protocol in compliance with the Animal Welfare Act, PHS Policy, and other federal statutes and regulations relating to animals and experiments involving animals. The facility where this research was conducted is accredited by the Association for Assessment and Accreditation of Laboratory Animal Care, International and adheres to principles stated in the Guide for the Care and Use of Laboratory Animals, National Research Council, 2011.

Female C57BL/6 mice (6–14 weeks) (Jackson Laboratories) were challenged via the intraperitoneal route (I.P.) with 1,000 plaque forming units (pfu) (~30,000 LD_50_) mouse-adapted EBOV (maEBOV). Mice were treated one-day post-challenge I.P. with 200 μg of antibody or PBS. Mice were observed daily for clinical signs of disease and lethality. Daily observations were increased to a minimum of twice daily while mice were exhibiting signs of disease. Moribund mice were humanely euthanized on the basis of IACUC- approved criteria.

Male and female Type 1 IFN α/β receptor knockout mice (Type 1 IFNα/β R^−/−^) purchased from Jackson Laboratory (4–14 weeks of age) were utilized for SUDV challenge experiments. Mice were challenged I.P. with a target dose of 1,000 pfu (~30,000 LD_50_) of wild-type SUDV and treated I.P. with 500 μg of indicated mAb at day +1 and +4.

Surviving mice from both experiments were rechallenged I. P. with target dose of 1,000 pfu of either maEBOV or wild-type SUDV with no mAb treatment provided. Following all challenges mice were observed daily for clinical signs of disease and lethality. Daily observations were increased to a minimum of twice daily while mice were exhibiting signs of disease. Moribund mice were humanely euthanized on the basis of IACUC- approved criteria.

Statistical comparisons of survival probability for both EBOV and SUDV challenges were made using the Fisher exact test with Bonferroni correction.

## Additional Information

**How to cite this article**: Frei, J. C. *et al.* Bispecific Antibody Affords Complete Post-Exposure Protection of Mice from Both Ebola (Zaire) and Sudan Viruses. *Sci. Rep.* **6**, 19193; doi: 10.1038/srep19193 (2016).

## Supplementary Material

Supplementary Information
