# Supplementary material for: Bispecific Antibody Affords Complete Post-Exposure Protection of Mice from Both Ebola (Zaire) and Sudan Viruses
Source: Sci Rep. 2016 Jan 13;6:19193. doi: 10.1038/srep19193 (PMC4725817; doi:10.1038/srep19193)
Supplement: Supplementary Information [file srep19193-s1.pdf]

Supplementary Information for:

Bispecific Antibody Affords Complete Post-Exposure Protection of Mice from Both Ebola (Zaire) and Sudan Viruses

Julia C. Frei,[1]* Elisabeth K. Nyakatura,[1]* Samantha E. Zak,[2]* Russell R. Bakken,[2] Kartik Chandran,[3] John M. Dye,[2] and Jonathan R. Lai[1]

[1] Department of Biochemistry, Albert Einstein College of Medicine, 1300 Morris Park Ave., Bronx, NY 10461. [2] Virology Division, United States Army Medical Research Institute of Infectious Disease, 1425 Porter Street, Fort Detrick, MD 21702. [3] Department of Microbiology and Immunology, Albert Einstein College of Medicine, 1300 Morris Park Ave., Bronx, NY 10461.

* These authors contributed equally to this work.

Correspondence and requests for materials should be addressed to J. M. D. (john.m.dye1.civ@mail.mil) or J. R. L. (jon.lai@einstein.yu.edu)

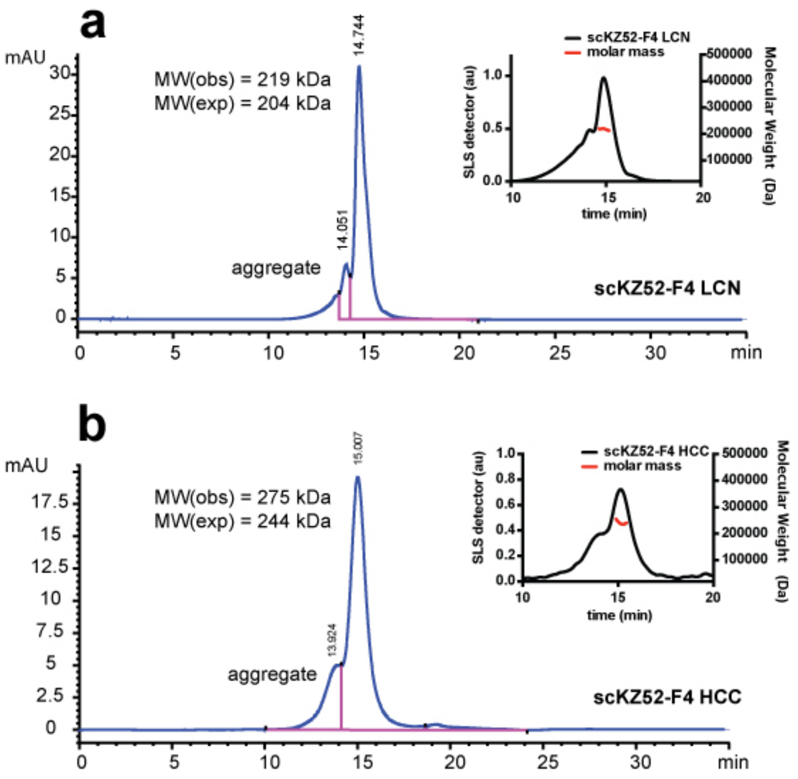

Supplementary Figure 1. Size exclusion chromatography-multiangle light scattering (SEC-MALS) analysis of Bis-mAbs. scKZ52-F4 LCN (a) and scKZ52-F4 HCC (b) were analyzed on an Agilent BioSec 5 in 150 mM HEPES buffer, pH 7.4, 200 mM NaCl. Absorbance was monitored at 280 nm. Molecular weight estimates from MALS shown in inset.

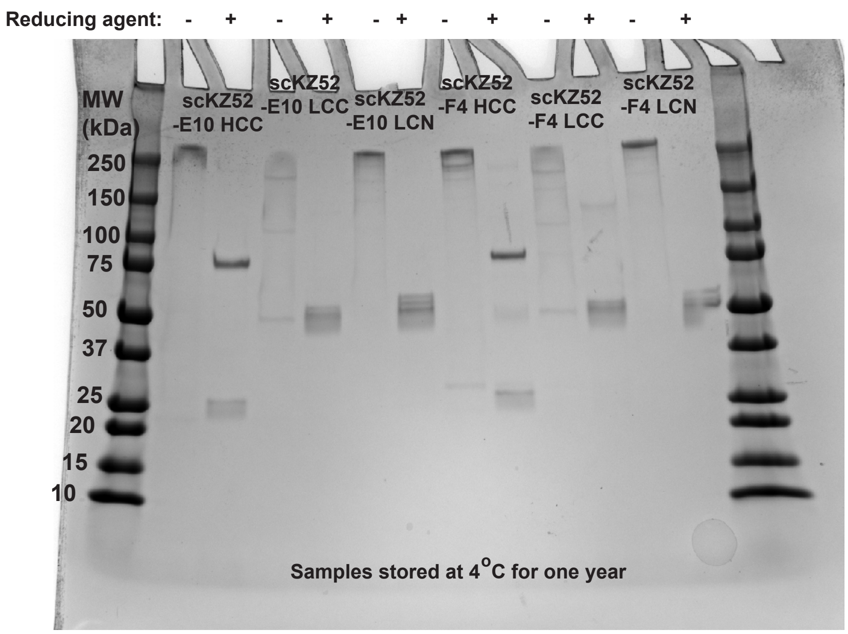

Supplementary Figure 2. SDS-PAGE analysis of Bis-mAbs after storage at 4 ºC for one year.

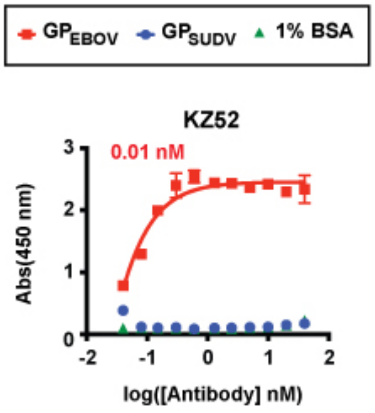

Supplementary Figure 3. ELISA of KZ52 IgG for GP and 1% BSA control.

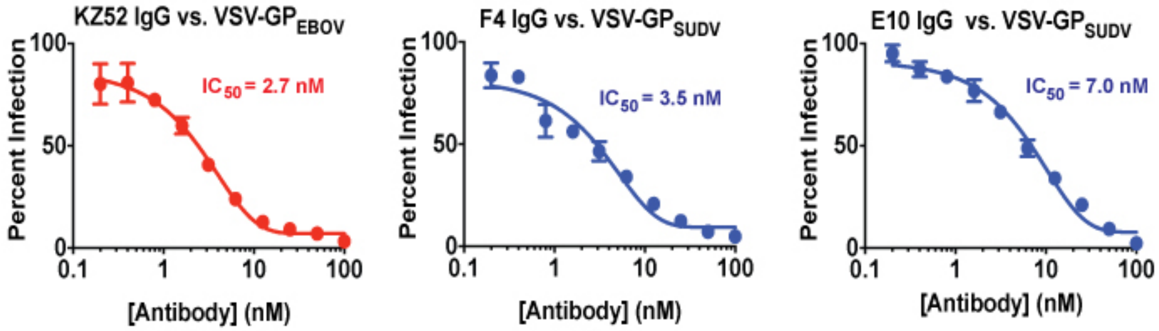

Supplementary Figure 4. Neutralization of VSV-GP by monospecific mAbs KZ52 (EBOV), F4 and E10 (SUDV)

Supplementary Table 1. Single-phase bioloayer interferometry (BLI) analysis of scKZ52-F4.

Analyte	k_a (M^{-1} sec^{-1})	k_d (sec^{-1})	K_D (nM)
GP_{EBOV}	$(3.7 \pm 0.1) \times 10^{3}$	$(8.7 \pm 0.3) \times 10^{-4}$	240 ± 8
GP_{SUDV}	$(6.4 \pm 0.1) \times 10^{4}$	$(1.1 \pm 0.01) \times 10^{-2}$	170 ± 3
